# Prevalence of Hypovitaminosis D and Its Association with Motor Impairment in Spinal Muscular Atrophy

**DOI:** 10.3390/nu18193155

**Published:** 2026-09-24

**Authors:** Dallianny Paulino Martins de Andrade, Antônio Vicente Dias de Andrade, Paloma Katlheen Moura Melo, Thais Araújo Barbosa, Dyego Luiz Pereira Lima de Melo e Silva, José Rodolfo Lopes de Paiva Cavalcanti, Epitácio Leite Rolim Filho

**Affiliations:** 1 Graduate Program in Surgery, Federal University of Pernambuco (UFPE), Recife 50670-901, PE, Brazil; dalliannymartins@gmail.com (D.P.M.d.A.); thaisaraujobarbosa@outlook.com (T.A.B.); dyegoluiz.net@gmail.com (D.L.P.L.d.M.e.S.); filhorolim@gmail.com (E.L.R.F.); 2 Laboratory of Experimental Neurology, Department of Biomedical Sciences, State University of Rio Grande do Norte, Mossoró 59607-360, RN, Brazil; dr_vicent@hotmail.com (A.V.D.d.A.); rodolfolopes@uern.br (J.R.L.d.P.C.)

**Keywords:** spinal muscular atrophy, vitamin D, neuromuscular diseases

## Abstract

**Background/Objectives:** In recent years, the role of vitamin D in the etiology of central and peripheral neurological diseases has been widely investigated, and evidence suggests benefits of vitamin D replacement therapy. However, few studies have evaluated the relationship between serum vitamin D levels and patients with spinal muscular atrophy (SMA). Therefore, this study aimed to analyze serum vitamin D levels in patients with SMA and their relationship with disease type, degree of motor impairment, and the presence of musculoskeletal deformities. **Methods:** This was a cross-sectional analytical study conducted with patients diagnosed with SMA who were treated at a Rare Diseases Reference Service in Recife, Pernambuco, Brazil. Serum vitamin D levels and their association with clinical characteristics of the disease, including SMA type, motor impairment, and the presence of musculoskeletal deformities were evaluated. Motor impairment was classified according to functional capacity, and an ordinal score was used for correlation analysis. **Results:** The sample consisted of 36 patients with SMA. The mean serum 25(OH)D concentration was 28.95 ± 10.28 ng/mL, with values ranging from 8.3 to 49.4 ng/mL. Based on the predefined cutoff of 30 ng/mL, 58.3% of participants had serum 25(OH)D concentrations below 30 ng/mL, whereas 41.7% had concentrations ≥30 ng/mL. Serum 25(OH)D concentrations differed significantly across motor function groups (*p* = 0.002). However, correlation analysis showed a weak and non-significant correlation between motor impairment and serum 25(OH)D concentrations (Spearman r = −0.136; *p* = 0.431; (*n *= 36). An association between hypovitaminosis D and the presence of musculoskeletal deformities was also observed. **Conclusions:** Hypovitaminosis D showed a high prevalence in patients with SMA. Although serum 25(OH)D concentrations differed significantly across motor function groups, the correlation between motor impairment and serum 25(OH)D concentrations was weak and non-significant. These findings reinforce the importance of monitoring serum vitamin D levels and adopting clinical and nutritional follow-up strategies in this population.

## 1. Introduction

In recent years, the role of vitamin D in the etiology of central and peripheral neurological diseases, both acute and chronic, has been increasingly investigated. Evidence also suggests potential benefits of vitamin D replacement therapy in these conditions. However, few studies have evaluated the relationship between serum vitamin D levels and patients with spinal muscular atrophy (SMA) [1,2].

SMA is a rare, progressive, and potentially fatal genetic disorder that affects the lower motor neurons of the spinal cord, leading to degeneration of the cells responsible for skeletal muscle innervation [2]. This condition results in progressive muscle weakness and atrophy, typically with onset in childhood, although late-onset forms have also been described. SMA is considered one of the leading genetic causes of infant mortality, with an estimated prevalence of approximately 1:10,000 live births and a carrier frequency of about 1 in 50 individuals [3].

The impact of SMA on motor function is substantial, requiring comprehensive and multidisciplinary care, including rehabilitation, orthopedic, and nutritional management [4].

In recent years, advances in the genetic understanding of SMA and the development of disease-modifying therapies have significantly improved its prognosis [5,6].

Vitamin D deficiency is considered a global public health concern and is often associated with inadequate cutaneous synthesis [7]. Experimental studies using animal models have demonstrated that vitamin D may promote myelination following peripheral nerve injury and reduce neuronal damage [8,9]. Conversely, deficiency of this vitamin may lead to hyperinnervation and hypersensitivity of nerve fibers, potentially increasing pain perception in various pathological conditions [1].

In addition, some medications investigated for the management of SMA, such as valproic acid, may interfere with the absorption of micronutrients, including vitamin D. Reduced levels of this vitamin may increase the risk of osteoporosis and fractures in children with SMA, highlighting the importance of careful monitoring of nutritional status and bone health in this population [10,11].

Therefore, this study aimed to evaluate serum vitamin D levels in patients with spinal muscular atrophy in order to contribute to a better understanding of the role of this micronutrient in the context of the disease.

## 2. Materials and Methods

### 2.1. Study Design and Participants

This was a cross-sectional analytical study conducted among patients with spinal muscular atrophy (SMA). Patients were recruited from the Rare Diseases Reference Service (RARUS), located in Recife, Pernambuco, Brazil. All participants had a previously established diagnosis of SMA.

The study protocol was approved by the Ethics Committee of Hospital Getúlio Vargas of Pernambuco (approval number: 7365059). Written informed consent and assent were obtained from the participants or their legal guardians, as applicable, prior to data collection. Clinical and demographic information was obtained through structured questionnaires and review of medical records. Samples were collected at the time of study enrollment.

### 2.2. Eligibility Criteria

Patients with a confirmed diagnosis of SMA who had not received vitamin D supplementation within the previous six months were eligible for inclusion.

Patients with systemic diseases that could interfere with the study results, as well as those diagnosed with other neuropathies or inflammatory syndromes, were excluded.

### 2.3. Biochemical Assessment

Serum 25-hydroxyvitamin D [25(OH)D] concentrations were measured to assess vitamin D status. For statistical analysis, participants were classified into two groups according to serum 25(OH)D concentrations: <30 ng/mL and ≥30 ng/mL.

The cutoff point of 30 ng/mL was adopted based on recommendations from the Brazilian Society of Clinical Pathology/Laboratory Medicine (SBPC/ML) and the Brazilian Society of Endocrinology and Metabolism (SBEM), which consider concentrations between 30 and 60 ng/mL to be recommended for individuals belonging to risk groups. Considering that the study population consisted of patients with SMA, a neuromuscular disease associated with substantial motor and musculoskeletal impairment, 30 ng/mL was established as the cutoff point for the dichotomous analysis.

For laboratory assessment, 10 mL of venous blood was collected between 8:00 and 10:00 a.m. Serum was used as the biological material for vitamin D determination. Samples were sent for analysis to a+ Medicina Diagnóstica, part of Grupo Fleury, located at Amaro Bezerra Street, 489, Derby, Recife, Pernambuco, Brazil. As the measurements were performed as part of routine laboratory procedures, the samples were not subjected to prolonged storage specifically for research purposes. Serum 25(OH)D concentrations were determined using a competitive electrochemiluminescence immunoassay.

Blood samples were collected by trained healthcare professionals, and biochemical analyses were performed in a certified clinical laboratory.

### 2.4. Clinical Interview and Data Collection

Information regarding medical history and clinical characteristics was obtained through interviews and review of medical records. The collected data included family medical history, presence of allergies, medication use, laboratory results, and findings from physical examination, including growth parameters and the presence of musculoskeletal deformities.

### 2.5. SMA Classification

The clinical classification of SMA was based on age at symptom onset and the highest motor milestone achieved. Individual participant characteristics, including sex, age at symptom onset, age at diagnosis, SMA type, and serum 25(OH)D concentration, are presented in Table 1.

### 2.6. Statistical Analysis

Statistical analyses were performed using Statistica software version 13.3 (StatSoft, Tulsa, OK, USA). Continuous variables were presented as mean ± standard deviation (SD), while categorical variables were expressed as absolute numbers and percentages.

Data distribution was assessed using the Shapiro–Wilk test. Depending on data distribution and the number of groups compared, the Mann–Whitney U test, chi-square test, or Kruskal–Wallis test with post hoc median test were applied.

For the correlation analysis, the degree of motor impairment was converted into a numerical ordinal variable. Progressively higher values were assigned to the functional categories according to the increasing severity of motor impairment, with higher values representing greater functional impairment: 1 = normal; 2 = ambulates without support; 3 = ambulates with support; 4 = cervical and trunk control; 5 = cervical control only; and 6 = no cervical control. This ordinal variable was used to assess the correlation with serum 25(OH)D concentrations. Associations between variables were analyzed using the Spearman correlation coefficient, and 95% confidence intervals (95% CI) were calculated when appropriate. Statistical significance was set at *p* < 0.05.

## 3. Results

### 3.1. Sample Characterization

Initially, 72 patients were selected to participate in this study. Of these, 28 were receiving oral vitamin D supplementation and were excluded according to the predefined exclusion criteria. Among the remaining 44 patients, 8 did not undergo the requested laboratory tests. Therefore, the final sample consisted of 36 patients diagnosed with spinal muscular atrophy (SMA) treated at the Rare Diseases Reference Service in Recife, Pernambuco, Brazil. The clinical and laboratory characteristics of the participants are presented in Table 2.

Regarding SMA classification, patients were distributed as follows: SMA type I (25.0%), type II (38.9%), type III (33.3%), and type IV (2.8%). All patients included in this study (100%) reported daily sun exposure for approximately 20 min in the morning before 8:00 a.m. Treatment with nusinersen (Spinraza) was reported in 61.1% of patients, while 38.9% were not receiving this medication. Regarding motor status, 11.1% of patients presented no motor impairment, 8.3% were able to walk without support, 8.3% walked with support, 47.2% had cervical and trunk control, 8.3% had only cervical control, and 16.7% had no cervical control. Musculoskeletal deformities were identified in 58.3% of the patients. Among the most frequent alterations were deformities of the hip (47.2%), knee (41.7%), spine (38.9%), and feet (16.7%), as well as deformities involving the shoulders, wrists, hands, and thorax (2.8%).

### 3.2. Vitamin D Levels in the Study Sample

The mean serum 25(OH)D concentration in the sample was 28.95 ± 10.28 ng/mL, with values ranging from 8.3 to 49.4 ng/mL. Based on the predefined cutoff of 30 ng/mL, 21 participants (58.3%) had serum 25(OH)D concentrations below 30 ng/mL, whereas 15 participants (41.7%) had concentrations ≥30 ng/mL. These findings indicate a high prevalence of serum 25(OH)D concentrations below the predefined cutoff in the studied sample (Table 2).

### 3.3. Relationship Between Vitamin D Levels and Motor Impairment

Analysis of serum 25(OH)D concentrations according to the degree of motor impairment showed that patients with greater functional dependence tended to present lower 25(OH)D concentrations. Motor impairment was classified according to the functional capacity of the patients, and serum 25(OH)D concentrations were compared among the different mobility groups. Comparison between mobility groups revealed a statistically significant difference in serum 25(OH)D concentrations across the different levels of motor function (*p* = 0.002). When the relationship between these variables was evaluated through correlation analysis, a weak and non-significant correlation was observed between the degree of motor impairment and serum 25(OH)D concentrations (Spearman’s r = −0.136; *p* = 0.431; n = 36) (Figure 1).

### 3.4. Relationship Between Vitamin D Levels and Musculoskeletal Deformities

Musculoskeletal deformities were identified in 58.3% of the patients. When musculoskeletal deformities were analyzed as an overall binary variable (presence vs. absence of any deformity), an association with serum 25(OH)D concentrations was observed (*p* = 0.014). However, when individual anatomical deformities were analyzed separately, none reached statistical significance (Table 3). Among the specific anatomical sites, patients with hip deformities tended to have lower serum 25(OH)D concentrations compared with those without hip deformities, although this difference did not reach statistical significance (*p* = 0.057). Serum 25(OH)D concentrations according to the presence of specific musculoskeletal deformities are presented in Table 3.

## 4. Discussion

The present study analyzed the prevalence of vitamin D deficiency in patients with spinal muscular atrophy (SMA), investigating factors associated with hypovitaminosis D and its possible relationship with the clinical severity of the disease. The findings reinforce the high prevalence of hypovitaminosis D in this population, even among patients who reported daily sun exposure, highlighting the need for specific strategies for monitoring and appropriate management of serum 25(OH)D concentrations in individuals with SMA [12,13,14,15].

In this study, 21 participants (58.3%) had serum 25(OH)D concentrations below 30 ng/mL, whereas 15 (41.7%) had concentrations ≥30 ng/mL. This finding indicates a high prevalence of serum 25(OH)D concentrations below the predefined cutoff in the studied population and is consistent with reports describing a high frequency of hypovitaminosis D among patients with neuromuscular diseases [12]. Previous studies suggest that individuals with SMA have an increased risk of vitamin D deficiency due to factors such as reduced mobility, lower sun exposure, restrictive diets, and, in some cases, prolonged corticosteroid use. In the present sample, patients with musculoskeletal deformities had significantly lower serum 25(OH)D concentrations [12,13,16].

The high frequency of serum 25(OH)D concentrations below 30 ng/mL observed in this study suggests that a substantial proportion of patients with SMA may have inadequate vitamin D status. This finding reinforces the importance of periodic monitoring of serum 25(OH)D concentrations and the implementation of appropriate supplementation strategies when clinically indicated in order to prevent complications associated with inadequate vitamin D status, such as impaired bone health, muscle weakness, and increased risk of fractures [12,13,17].

However, the correlation analysis based on the ordinal motor impairment score did not demonstrate a statistically significant association between motor impairment and serum 25(OH)D concentrations (Spearman’s r = −0.136; *p* = 0.431). Patients with more severe motor limitations, such as those presenting only cervical control or no cervical control, showed lower mean serum 25(OH)D concentrations when compared with patients who were able to walk with or without support. These findings indicate that serum 25(OH)D concentrations differed across motor function categories; however, the correlation analysis did not demonstrate a statistically significant monotonic association between the ordinal motor impairment score and serum 25(OH)D concentrations [14,18].

Reduced mobility in these patients may limit sun exposure, which is the main pathway for vitamin D synthesis in the human body. The literature indicates that cutaneous synthesis of vitamin D depends directly on ultraviolet B (UVB) radiation, and insufficient sun exposure is one of the main risk factors for hypovitaminosis D. In individuals with SMA, prolonged immobility may contribute to inadequate vitamin D status, increasing the risk of bone fragility and osteoporosis. However, in the present study, all patients reported daily sun exposure for approximately 20 min in the morning, suggesting that other factors may also contribute to hypovitaminosis D in this population [12,13,14].

In addition to sun exposure, dietary intake of vitamin D is also an important factor in maintaining adequate serum levels. Patients with SMA frequently present dietary restrictions due to swallowing difficulties, which may compromise the intake and absorption of essential nutrients [12,13,15].

In the present study, patients who used alternative feeding routes, such as nasogastric tube (NGT) or gastrostomy (GTT), showed a higher prevalence of vitamin D deficiency, although this association did not reach statistical significance (*p* = 0.355). This finding suggests that diet may not be adequately meeting vitamin D requirements in these patients, highlighting the importance of individualized nutritional assessment and consideration of oral or enteral supplementation when necessary [2,12,13].

Analysis of the relationship between serum 25(OH)D concentrations and the clinical type of SMA showed that patients with SMA type I had the lowest mean serum 25(OH)D concentrations, whereas patients with SMA type IV presented the highest levels. Despite this trend, no statistically significant association was observed between SMA type and serum 25(OH)D concentrations (*p* = 0.883). On the other hand, an association between hypovitaminosis D and the presence of musculoskeletal deformities was observed (*p* = 0.014), indicating that patients with deformities presented significantly lower serum 25(OH)D concentrations [12,13,16].

The relationship between hypovitaminosis D and bone fragility is well documented in the literature. Vitamin D deficiency compromises calcium and phosphorus absorption, leading to bone demineralization, increased risk of fractures, and the development of skeletal deformities [19]. In the present study, no statistically significant association was observed between SMA type and serum 25(OH)D concentrations, whereas the presence of musculoskeletal deformities was associated with lower serum 25(OH)D concentrations. These findings should be interpreted cautiously given the cross-sectional design and the relatively small sample size.

The results of this study have important implications for the clinical management of patients with SMA. The high prevalence of serum 25(OH)D concentrations below 30 ng/mL observed suggests that periodic assessment of serum 25(OH)D concentrations should be incorporated into the clinical follow-up of these patients. Furthermore, strategies including nutritional supplementation and guidance on appropriate sun exposure may help prevent bone and muscle complications associated with inadequate vitamin D status.

This study has some limitations that should be considered when interpreting the results. First, this was a cross-sectional study conducted in a single center, which may limit the generalization of the findings to other SMA populations. In addition, the sample size was relatively small, a common characteristic in studies involving rare diseases. Another relevant limitation is that additional factors that may influence serum vitamin D levels, such as detailed dietary habits, seasonal variation in sun exposure, and levels of physical activity, were not extensively evaluated.

Overall, the results of this study demonstrate that serum 25(OH)D concentrations below 30 ng/mL were highly prevalent in patients with spinal muscular atrophy, even among those who reported regular sun exposure. Although differences in serum 25(OH)D concentrations were observed across motor function categories, the correlation between the ordinal motor impairment score and serum 25(OH)D concentrations was weak and non-significant. The association observed between musculoskeletal deformities and lower serum 25(OH)D concentrations further supports the importance of systematic monitoring of vitamin D status and musculoskeletal health in patients with SMA.

## 5. Conclusions

This study demonstrated a high prevalence of serum 25(OH)D concentrations below 30 ng/mL in patients with spinal muscular atrophy, with lower concentrations observed among patients with musculoskeletal deformities. Although differences in serum 25(OH)D concentrations were observed across motor function categories, the correlation between motor impairment and serum 25(OH)D concentrations was weak and non-significant. These findings indicate that hypovitaminosis D represents a frequent condition in this population and may be associated with negative impacts on bone and muscle health.

Therefore, the results highlight the importance of periodic monitoring of serum 25(OH)D concentrations in patients with SMA, and implementation of clinical and nutritional follow-up strategies that include guidance on appropriate sun exposure and evaluation of the need for supplementation. Such measures may contribute to the prevention of musculoskeletal complications and support the maintenance of bone and muscle health in these patients.

## Figures and Tables

**Figure 1:**
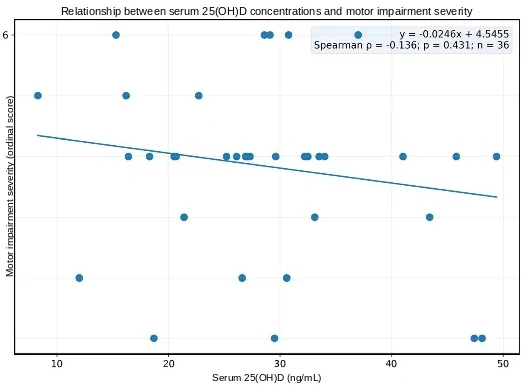
Correlation between serum 25(OH)D concentrations and degree of motor impairment in patients with spinal muscular atrophy (SMA). The analysis demonstrated a weak and non-significant correlation between serum 25(OH)D concentrations and motor impairment (Spearman’s r = −0.136; *p* = 0.431; n = 36). Source: authors’ own research data (2025). The dots represent individual participants, and the line represents the linear trend of the data.

**Table 1:** Individual characteristics of participants and serum 25(OH)D concentrations.

UPN	Sex	Age at Symptom Onset	Age at Diagnosis	SMA Type	25(OH)D (ng/mL)
UPN 01	Female	1 month	1 month	SMA III	29.50
UPN 02	Female	1 month	1 year	SMA I	30.76
UPN 03	Female	3 months	4 months	SMA I	37.00
UPN 04	Male	1 month	2 months	SMA I	37.00
UPN 05	Male	3 months	7 months	SMA I	28.60
UPN 06	Female	15 days	2 months	SMA I	26.90
UPN 07	Male	2 months	4 months	SMA I	29.10
UPN 08	Female	3 months	1 year 6 months	SMA I	22.70
UPN 09	Male	15 days	4 months	SMA I	15.30
UPN 10	Female	9 months	4 years	SMA II	45.80
UPN 11	Male	1 year	1 year 6 months	SMA II	43.40
UPN 12	Female	7 months	2 years	SMA II	8.30
UPN 13	Male	6 months	1 year 6 months	SMA II	20.70
UPN 14	Female	9 months	1 year 6 months	SMA III	21.40
UPN 15	Male	9 months	2 years	SMA II	34.00
UPN 16	Female	9 months	4 years	SMA II	18.30
UPN 17	Female	6 months	1 year	SMA II	26.10
UPN 18	Male	11 months	2 years	SMA II	25.20
UPN 19	Male	2 years	2 years	SMA III	30.60
UPN 20	Male	1 year	3 years	SMA III	16.40
UPN 21	Male	12 years	15 years	SMA III	48.10
UPN 22	Male	10 years	10 years	SMA III	47.40
UPN 23	Female	2 years	7 years	SMA III	32.50
UPN 24	Male	11 months	2 years	SMA II	27.30
UPN 25	Male	1 year 7 months	2 years	SMA II	33.50
UPN 26	Male	15 years	15 years	SMA III	26.61
UPN 27	Female	2 years	12 years	SMA III	32.20
UPN 28	Male	1 year	7 years	SMA III	27.07
UPN 29	Male	6 months	2 years	SMA II	29.60
UPN 30	Male	15 years	18 years	SMA III	12.00
UPN 31	Female	17 years	19 years	SMA IV	33.10
UPN 32	Male	1 year	2 years	SMA II	20.50
UPN 33	Male	11 months	1 year 6 months	SMA II	49.40
UPN 34	Female	10 months	4 years	SMA II	41.01
UPN 35	Male	4 months	1 year	SMA I	16.20
UPN 36	Male	14 years	18 years	SMA III	18.70

UPN = unique participant number; SMA = spinal muscular atrophy; 25(OH)D = 25-hydroxyvitamin D. Source: Authors’ own research data (2025).

**Table 2:** Clinical classification of spinal muscular atrophy (SMA) and serum 25(OH)D concentrations according to SMA type.

SMA Type	Typical Age at Onset	Highest Motor Milestone	n	Mean 25(OH)D	SD	Median	Minimum	Maximum
SMA I	<6 months	Unable to sit without support	9	27.06	7.85	28.60	15.30	37.00
SMA II	6–18 months	Able to sit but unable to walk independently	14	30.22	11.72	28.45	8.30	49.40
SMA III	>18 months	Able to walk independently	12	28.54	11.07	28.29	12.00	48.10
SMA IV	Adulthood	Able to walk independently	1	33.10	0.00	33.10	33.10	33.10

SMA = spinal muscular atrophy; 25(OH)D = 25-hydroxyvitamin D; SD = standard deviation. Source: authors’ own research data (2025).

**Table 3:** Descriptive statistics of serum 25(OH)D concentrations in participants (n = 36) according to the presence of specific musculoskeletal deformities.

Musculoskeletal Deformities	Group	n	Vitamin D			*p*-Value
			Mean ± SD	Median	Min–Max.	
Hip	Yes	17	25.51 ± 7.92	27.07	8.30–41.01	0.057
	No	19	32.02 ± 11.33	30.76	12.0–49.40	
Knee	Yes	15	27.42 ± 8.46	29.10	8.30–41.01	0.461
	No	21	30.04 ± 11.48	27.30	12.0–49.40	
Foot	Yes	06	25.43 ± 7.44	23.95	16.20–37.0	0.365
	No	30	29.65 ± 10.72	29.30	8.30–49.40	
Spine	Yes	14	27.08 ± 7.57	26.25	16.20–41.01	0.391
	No	22	30.14 ± 11.69	29.55	8.30–49.40	
Shoulder	Yes	01	16.40 ± 0.0	16.40	16.40–16.40	-
	No	35	29.31 ± 10.19	29.10	8.30–49.40	
Elbow	Yes	0	-	-	-	-
	No	36	28.95 ± 10.28	28.85	8.30–49.40	
Wrist	Yes	01	16.40 ± 0.0	16.40	16.40–16.40	-
	No	35	29.31 ± 10.19	29.10	8.30–49.40	
Thoracic region	Yes	01	15.30 ± 0.0	15.30	15.30–15.30	-
	No	35	29.34 ± 10.15	29.10	8.30–49.40	

Statistical significance was set at *p* < 0.05. 25(OH)D = 25-hydroxyvitamin D; SD = standard deviation. Source: authors’ own research data (2025).

## Data Availability

The data presented in this study are available from the corresponding author upon reasonable request.
